# Which factors modulate spontaneous motor tempo? A systematic review of the literature

**DOI:** 10.3389/fpsyg.2023.1161052

**Published:** 2023-10-18

**Authors:** Anaïs Desbernats, Elodie Martin, Jessica Tallet

**Affiliations:** ToNIC, Toulouse NeuroImaging Center, Université de Toulouse, Inserm, UPS, Toulouse, France

**Keywords:** SMT, rhythm, intertap interval, intra-individual, inter-individual, variability, frequency

## Abstract

Intentionally or not, humans produce rhythmic behaviors (e.g., walking, speaking, and clapping). In 1974, Paul Fraisse defined rhythmic behavior as a periodic movement that obeys a temporal program specific to the subject and that depends less on the conditions of the action (p. 47). Among spontaneous rhythms, the spontaneous motor tempo (SMT) corresponds to the tempo at which someone produces movements in the absence of external stimuli, at the most regular, natural, and pleasant rhythm for him/her. However, intra- and inter-individual differences exist in the SMT values. Even if several factors have been suggested to influence the SMT (e.g., the age of participants), we do not yet know which factors actually modulate the value of the SMT. In this context, the objectives of the present systematic review are (1) to characterize the range of SMT values found in the literature in healthy human adults and (2) to identify all the factors modulating the SMT values in humans. Our results highlight that (1) the reference value of SMT is far from being a common value of 600 ms in healthy human adults, but a range of SMT values exists, and (2) many factors modulate the SMT values. We discuss our results in terms of intrinsic factors (in relation to personal characteristics) and extrinsic factors (in relation to environmental characteristics). Recommendations are proposed to assess the SMT in future research and in rehabilitative, educative, and sport interventions involving rhythmic behaviors.

## 1. Introduction

Rhythm is an essential human component. “Rhythm is defined as the pattern of time intervals in a stimulus sequence” (Grahn, 2012, p. 586), and the tempo is the rate of the stimuli's onset within a regular sequence (Grahn, 2012). Early in life, rhythm is present in a large number of activities of daily life, such as walking, speaking, chewing, doing leisure activities (dancing, swimming, pedaling, playing a musical instrument, singing, clapping, etc.), or school activities (writing and reading). Some activities require producing a rhythm with a spontaneous tempo (e.g., writing, reading, chewing, walking, speaking, etc.), and some others require synchronizing with a rhythm produced by an external event (e.g., playing a musical instrument, singing, clapping, dancing, etc.). Those activities can have different rhythmic components. For example, speech generally shows a non-isochronous rhythmic structure, but other language skills, such as reading, may also show beat-based patterns (i.e., isochronous patterns based on equal time intervals; see Ozernov-Palchik and Patel, 2018). Writing seems to be linked to isochronous rhythmic production (Lê et al., 2020b), even if it is not yet well-known whether writing shows more beat- or non-beat-based processing. Other activities, such as tapping or clapping, are well-known to show isochronous patterns.

Rhythmic abilities are deficient in various populations, and nowadays, rehabilitative interventions based on rhythmic synchronization are used to improve motor control. This is the case for populations with neurological diseases (e.g., Parkinson's disease, stroke, and cerebral palsy; see Braun Janzen et al., 2021), rare diseases or conditions (Launay et al., 2014; Bégel et al., 2017, 2022a; Tranchant and Peretz, 2020), or neurodevelopmental disorders (e.g., dyslexia, developmental coordination disorder, and attention deficit and hyperactivity disorder; Puyjarinet et al., 2017; Bégel et al., 2018, 2022b; Lê et al., 2020a; Blais et al., 2021; Daigmorte et al., 2022). In this context, participants are required to synchronize their movements to an external rhythm, usually with an auditory metronome, to regulate the speed of their gait or manual or verbal responses. The ability to synchronize with an external rhythm is particularly studied during sensorimotor synchronization tasks that consist of the “coordination of a rhythmic movement with an external rhythm” (Repp and Su, 2013, p. 1). The tempo and the sensory modality of the external rhythmic stimuli can modulate the performance of sensorimotor synchronization (see Repp, 2005; Repp and Su, 2013 for extensive reviews of the literature). Sensorimotor synchronization is less accurate and stable when the tempo is slower (Drewing et al., 2006; Repp and Su, 2013) and slower than the spontaneous motor tempo (SMT; Varlet et al., 2012). SMT is the rhythm at which a person produces movements in the absence of stimuli at his/her own most regular, natural, and pleasant rate. Hence, the tempo of the external rhythm has to be adapted to the actual tempo of the participants. Recent studies individualize the parameters of the intervention by adapting the tempo of the metronome to be synchronized (Benoit et al., 2014; Dalla Bella et al., 2017; Cochen De Cock et al., 2021; Frey et al., 2022). This is done by measuring the individual's SMT before an intervention. Rehabilitation is then performed with music at either ±10% of this tempo. Therefore, it seems interesting to evaluate rhythmic abilities, especially spontaneous motor tempo (SMT), to individualize learning and rehabilitation.

It is usually admitted in the pioneering work of Paul Fraisse that the most representative reference value of the spontaneous motor tempo (SMT) is 600 ms in healthy human adults (Fraisse, 1974). However, a growing body of literature about SMT suggests that this value is not universal. Fraisse himself pointed out that, even if the SMT is supposed to be relatively stable in one individual, inter-individual differences are more important and could be related to the instructions, the material of measurement, the body position, the chronological and intellectual development, and the sensory deficits (Fraisse, 1974). Even if these factors have been tested in a few studies, to our knowledge, no updated review of the literature has been made to provide complete and recent knowledge on the range of SMT values in healthy human adults and the factors influencing them. For example, recent studies suggest that age is a major factor modulating the value of SMT. The review by Provasi et al. (2014a) focuses on the spontaneous (and induced) rhythmic behaviors during the perinatal period, with a special emphasis on the spontaneous rhythm of sucking, crying, and arm movements in newborns. The authors indicate that the SMT evolves from newborns to the elderly. Fast rhythmical movements of the arms have been identified in fetuses with a tempo of 3 or 4 movements per second (250–333 ms; Kuno et al., 2001), whereas a tempo of 450 ms has been found during drumming (Drake et al., 2000) or tapping (McAuley et al., 2006) in children around 4 years old and more. The value of the SMT is relatively fixed around 400 ms between 5 and 8 years, even if the variability of the SMT tends to decrease with age (Monier and Droit-Volet, 2019). The SMT is supposed to increase to achieve 600 ms in adulthood (Fraisse, 1974) and to slow down further with age to achieve 700–800 ms in the elderly (Vanneste et al., 2001). In the case of tempo produced with the mouth, the SMT of non-nutritive sucking is around 450 ms in neonates (Bobin-Bègue et al., 2006), whereas the spontaneous crying frequency is between 1,100 and 2,400 ms in newborns (Brennan and Kirkland, 1982). All these results suggest that the relationship between SMT and age is *not* general and linear. The effector producing the SMT could be a potential factor affecting the relationship between SMT and age.

Some studies focus on the SMT produced with the mouth in a quasi-rhythmic pattern during speech production and in an isochronous repetitive pattern during syllable rate production. The review of Poeppel and Assaneo (2020) reports that the temporal structure of speech “is remarkably stable across languages, with a preferred range of rhythmicity of 2–8 Hz” (125–500 ms; Poeppel and Assaneo, 2020, p. 322). One could suggest that this rhythm is faster than the rhythm supposed to be found in rhythmical movements of the arms (600 ms in adulthood, Fraisse, 1974). However, in the broader context of speech production, we cannot neglect the communicative aspect of speech. The audience for the speech could also influence the SMT (Leong et al., 2017). Thus, it is possible that, in addition to the age previously mentioned, not only the effector but also the communicative goal of the activity may influence the SMT.

Moreover, environmental factors are supposed to influence SMT values. In the review of Van Wassenhove (2022), it is suggested that the manipulation of external landmarks, such as the time of day, can modulate the endogenous temporal representation of time and, as a consequence, the SMT (Van Wassenhove, 2022).

In this context, the objectives of the systematic review are (1) to characterize the range of SMT values found in the literature in healthy human adults and (2) to identify all the factors modulating the SMT values in humans.

## 2. Materials and methods

We conducted a systematic review according to PRISMA recommendations (Preferred Reporting Items for Systematic Reviews and Meta-Analyses; Page et al., 2021).

### 2.1. Information sources and search strategy

Studies were identified by searching in the PubMed, Science Direct, and Web of Science databases. These databases were selected because they represent a broad spectrum of disciplines related to motor behavior. The final search was performed on 4 July 2022. There was no restriction on the year of publication; all articles present in the databases at this time point were searched. The search was first conducted in all languages, and then only English and French studies were selected for screening. As the term “spontaneous motor tempo” is not exclusively used, we searched a broad spectrum of synonyms for this term. Filters were also used to identify relevant research depending on the database (Table 1).

**Table 1:** Search strategy information.

	**PubMed**	**Science Direct**	**Web of Science**
Search equation	((spontaneous motor tempo) OR ((spontaneous OR self-paced OR internally-driven OR internal OR preferred OR internally-guided) AND (motor NOT locomotion NOT locomotor) AND (tempo OR rhythm OR rhythmic OR tapping OR (intertap interval))))	(‘human') AND ((‘spontaneous motor tempo') NOT (‘locomotion' OR ‘locomotor')	ALL = (human) AND (ALL = ((spontaneous motor tempo) OR ((spontaneous OR self-paced OR internally-driven OR internal OR preferred OR internally-guided) AND (motor NOT locomotion NOT locomotor) AND (tempo OR rhythm OR rhythmic OR tapping OR (intertap interval)))))
Applied filters	“Human” and “All type of documents”	“Review articles” and “Research articles”	“All type of documents”
Search results	1,225	1,141	813

### 2.2. Selection of studies and eligibility criteria

We only selected articles and reviews before screening by excluding congress papers, chapters, books, and theses. Reviews identified in databases were just used to find missing original articles about SMT, and they have not been included in the systematic review (reviews not included: Provasi et al., 2014a; Poeppel and Assaneo, 2020; Van Wassenhove, 2022).

For greater specificity in the selection of the studies, inclusion criteria were based on the PICO (population, intervention, comparator, and outcome) strategy (Table 2). For this, we selected studies carried out on human samples producing rhythmic tasks. A control factor or control group was identified as a comparator. Spontaneous motor tempo was identified as the Outcome. Moreover, we selected other exclusion criteria: (1) studies that did not present experimental data; (2) studies that did not present a SMT task (i.e., focusing only on sensorimotor synchronization or on perception of rhythmic stimuli); (3) studies that did not report data on SMT (a SMT task is produced by the participants, but variables studied assess, for example, brain data or relative phases); (4) studies that did not focus on intentional SMT (studies on cardiorespiratory rhythms like breath or heart rate); and (5) studies that focus on walking with displacement (locomotion). We excluded studies on locomotion because locomotion involves spatiotemporal regulation; however, we retained studies on walking on a treadmill because walking on a treadmill involves mainly temporal regulation.

**Table 2:** Description of the PICO strategy that was used.

**PICO strategy**	
**Description**	**Component**
Population	Human
Intervention	Rhythmic task
Comparator	Control factor or group
Outcomes	Spontaneous motor tempo

All titles and abstracts were screened by one researcher (AD), and if the articles fit the review criteria, they were read in full. The full-text eligibility assessment was conducted by two independent reviewers (AD and JT). Disagreements were resolved by a discussion according to the PICO strategy with a third researcher (EM).

### 2.3. Data collection process

For tabulation and extraction of data referring to the selected studies, Excel^®^ software spreadsheets were used. After screening the selected studies, we classified them into two categories, i.e., those measuring the SMT values (in general, as a prerequisite for a subsequent rhythmic sensorimotor synchronization task) and those examining the effect of factor(s) on the SMT values.

For studies measuring the SMT values, we extracted study characteristics, demographic variables, methodological variables, and outcome indicators from each study. The extracted characteristics included the authors, the year of publication, and the sample size. Demographic variables included sex, age, and laterality. Methodological variables included the instruction, the task, the effector(s), and the measurement recording. Outcome indicators included SMT values and their units. We finally convert all of the SMT values to milliseconds to be comparable and to provide a range of SMT values.

For studies about factor(s) modulating SMT values, we extracted study characteristics (first author and year of publication), methodological variables (task and effector(s)), and outcome indicators (factor(s) effects, their significance, and their direction on SMT values, i.e., on the mean or median and/or the standard deviation or coefficient of variation). Sometimes, we also extracted other information (e.g., subgroups and specific statistical analyses) to understand and interpret the results.

## 3. Results

**Figure 1:**
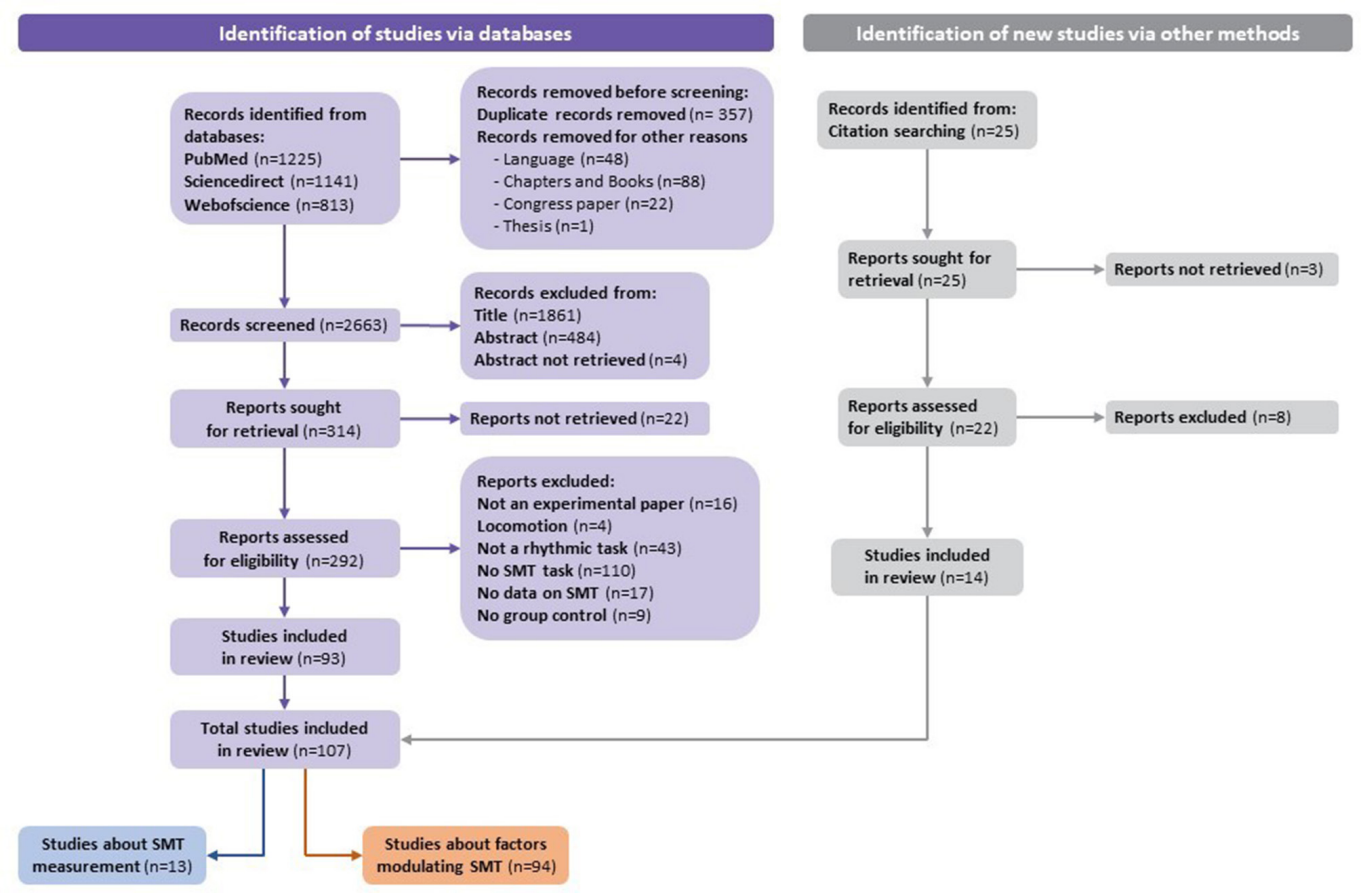
Flowchart of the identification of studies via databases.

A total of 3,179 studies were identified via databases. Before screening, 357 duplicates and 159 studies were removed (e.g., language, chapters and books, congress papers, or theses). According to the exclusion criteria, 2,349 studies were excluded based on the title or the abstract. After verifying the records left in full, according to the pre-established eligibility criteria, 93 studies from databases were included in the systematic review. Moreover, 14 out of 25 studies identified via citation searching were included. Finally, a total of 107 studies were included in the systematic review. Results from the process for selecting the included articles (following the recommendations of Page et al., 2021) are described in the flowchart (Figure 1).

In total, 13 studies provide a SMT value or a range of SMT values in healthy adults (Table 3). Our results reveal that the range of SMT values is from 333 to 3,160 ms. Notably, 94 studies measure the effect of the factor(s) on the SMT values (Table 4). We classified studies according to the type of factors modulating the SMT values: *intrinsic factors*, in relation to personal characteristics, and *extrinsic factors*, in relation to environmental characteristics. Concerning intrinsic factors, we have found studies investigating the effects of a pathology (*N* = 27), age (*N* = 16), the effector or the side (*N* = 7), the expertise or a predisposition (*N* = 7), and the genotype (*N* = 2). Concerning extrinsic factors, we have found studies investigating the effects of physical training (*N* = 10), external constraints (*N* = 7), observation training (*N* = 5), the time of testing (*N* = 4), the internal state (*N* = 3), the type of task (*N* = 5), and a dual task (*N* = 2).

**Table 3:** Summarized results of studies measuring SMT values (*N* = 13).

**References**	**Participants processed**		**Paradigm**					**SMT**					
	**Number of participants**	**Sex Age ±SD (years old) Laterality**	**Instruction**	**Task**	**Trial(s) (duration or intervals number)**	**Measurement recording**	**Effector**	**SMT values**			**Converted SMT values (in ms)**		**Coefficient of variation**
								**Mean, median or range**	**SD**	**Unit**	**Mean, median, or range**	**SD**	
Hattori et al. (2015)	6	2M 4F 27 ± N.S. Not reported	Not reported	Tapping	1 (30 times)	Intertap intervals	Fingers	333–505	12.6–23	ms	333–505	12.6–23	Not reported
Ruspantini et al. (2012)	11	Not reported Not reported Not reported	To periodically articulate the/pa/syllable, mouthing silently, at a self-paced, comfortable rate	Producing a syllable	Not reported	Syllable rate	Mouth/lips	2.1	0.5	Hz	476	200	Not reported
McPherson et al. (2018)	20	5M 15F 18–26 19 right-handed 1 left-handed	To hit the drum, sustaining a constant pulse at their own, naturally comfortable tempo	Drumming	10 (15 s each)	Beats per minute	Hand	62–122 (one at 189)	Not reported	bpm	492–968 (one at 317)	Not reported	Not reported
Rousanoglou and Boudolos (2006)	11	5M 6F 21.2 ± 0.5 (M) 21.3 ± 0.5 (F) Not reported	To perform two-legged hopping in place at their preferred hopping frequency	Hopping	2 (15 s each)	Duration of the hopping cycle	Legs	0.555	0.083	s	555	83	Not reported
Michaelis et al. (2014)	14	7M 7F 18–35 Right-handed	To tap a response key at whichever rate felt “most comfortable,” to keep a steady pace, and make the spaces between taps as even as possible	Tapping	4 (30 intertap intervals)	Intertap intervals	Finger	0.68	0.32	s	680	320	Not reported
Sidhu and Lauber (2020)	11	8M 3F 25.9 ± 3.8 Not reported	To cycle at a freely chosen cadence	Cycling on a cycle ergometer	1 (5 min)	Cadence	Legs	71.6	8.1	rpm	838	95	Not reported
Zhao et al. (2020)	21	13M 8F 26.2 ± 5.4 19 right-handed 2 left-handed	To perform rhythmic oscillatory movements at their preferred frequency (if he or she can do it all day long) with the amplitude of the participant's shoulder	Performing rhythmic oscillatory movements with a stick	1 (30 s)	Number of movement cycles	Hand	17–33	Not reported	no unit	909–1,765	Not reported	Not reported
De Pretto et al. (2018)	14	7M 7F 27.7 ± 3.1 Right-handed	To tap at their most natural pace, at a frequency they could maintain without mental effort, and for a long period of time	Tapping	3 (40 intertap intervals)	Intertap intervals	Finger	931	204	ms	931	204	5.6 ± 1.3%
Eriksson et al. (2000)	12	5M 7F 25–45 Not reported	Not reported	Opening and closing the jaw Chewing	2 (12 s each) 2 (12 s each)	Cycle time Cycle time	Jaw Jaw	2.43 0.86	0.86 0.16	s s	2,430 860	860 160	Not reported
Sotirakis et al. (2020)	20	Not reported 27.1 ± 9.15 Not reported	To perform voluntary postural sway cycles at their own self-selected amplitude and pace	Swaying	1 (20 cycles)	Cycle duration	Whole body	3,160	530	ms	3,160	530	Not reported
Malcolm et al. (2018)	16	11M 5F 25.6 ± 4.5 Right-handed	Not reported	Walking on a treadmill	Not reported	Speed walking	Legs	3.2–4.5	Not reported	km/h	Not convertible	Not reported	Not reported
LaGasse (2013)	12	Not reported 18–35 Not reported	To repeat the syllable/pa/at a comfortable and steady pace	Producing a syllable	7 (8 sequential repetitions)	Inter-responses interval	Mouth/lips	Not reported	Not reported	Not reported	Not reported	Not reported	Not reported
Zhao et al. (2017)	22	12M 10F 26.9 ± 6.6 Not reported	To tap at a constant and comfortable tempo	Tapping	6 (30 s each)	Not reported	Finger	Not reported	Not reported	Not reported	Not reported	Not reported	Not reported

The original SMT values reported were converted to milliseconds by the authors (A.D., E.M., and J.T.) to provide a range of SMT values in milliseconds: [333–3,160 ms].

**Table 4:** Summarized results of studies investigating the effects of factors on the SMT values (*N* = 94).

**References**	**Factors modulating the SMT**										
	**I. Intrinsic factors**										
	**1. Pathology**	**Significance**	**Direction of the effect (on mean or median of SMT)**	**Direction of the effect (on the SD or coefficient of variation of SMT)**	**Other factor(s)**	**Direction of the effect (on mean or median of SMT)**	**Direction of the effect (on the SD or coefficient of variation of SMT)**	**Interaction**	**Task(s)**	**Effector(s)**	**Other information**
Amrani and Golumbic (2020)	ADHD vs. Healthy adults	Yes	ADHD faster than Healthy adults	ADHD less stable than Healthy adults (within trial and across sessions)	/	/	/	/	Tapping on an electro-optic sensor	Finger	/
Byblow et al. (2002)	Parkinson's vs. Healthy elderly	Yes	Parkinson's is slower than Healthy elderly	Not found	Mode of coordination Side	Inphase faster than antiphase Not found	Not found Not found	No interaction	Producing pronation and supination movements	Forearm	/
Delevoye-Turrell et al. (2012)	Schizophrenia vs. Healthy adults	Yes	• Schizophrenia is slower than Healthy adults	• Schizophrenia is less stable than Healthy adults	/	/	/	/	Producing finger down and up rhythmic movements	Finger	/
	Ultra-High Risk vs. Healthy Younger adults	Yes	• Ultra-High Risk = Healthy Younger adults	• Ultra-High Risk less stable than Healthy young adults • Ultra-High Risk = Schizophrenia							
Flasskamp et al. (2012)	Parkinson's vs. Healthy elderly	Yes	Parkinson's faster than Healthy elderly	Parkinson's less stable than Healthy elderly	/	/	/	/	Producing a syllable	Mouth/lips	Subgroups of Parkinson's (Left-sided vs. Right-sided symptoms)
Frankford et al. (2021)	Stammerers vs. Healthy adults	No	Stammerers = Healthy adults	Stammerers = Healthy adults	/	/	/	/	Reading sentences	Mouth/lips	/
Häggman-Henrikson et al. (2002)	Whiplash-associated disorders vs. Healthy adults	Yes	Whiplash-associated disorders slower than Healthy adults	Not found	/	/	/	/	Chewing	Jaw	/
Horin et al. (2021)	Parkinson's vs. Healthy elderly	Yes	Parkinson's faster than Healthy elderly	Parkinson's = Healthy elderly	Effector	• Finger faster than Gait • Foot faster than Gait	• Finger = Gait • Foot = Gait	Interaction Pathology × Effector: Parkinson's faster than Healthy elderly for foot tapping	• Tapping on a keyboard key • Tapping on a pedal	• Finger • Foot	Other 5 m walking task
Keil et al. (1998)	Schizophrenia vs. Healthy adults	No	Schizophrenia = Healthy adults	Not found	Movement direction	Vertical faster than Horizontal	Not found	Not found	Bimanual coordination task	Fingers	Horizontal and vertical movements
Konczak et al. (1997)	Parkinson's vs. Healthy elderly	Yes	• Producing a syllable: Significant effect (no other information) • Tapping: Significant effect (no other information)	• Producing a syllable: Not found • Tapping: Not found	Task (Dual vs. Single)	• Producing a syllable: Significant effect (no other information) • Tapping: Not found	• Producing a syllable: Not found • Tapping: Not found	Not found	• Producing a syllable • Tapping on a table	• Mouth/lips • Finger	Subgroups of Parkinson's (With vs. Without hastening)
Kumai (1999)	2–3.5 vs. 3.6–4.5 vs. 4.6–5.5 vs. 5.6–6.11 vs. 7+ years of mental ages	No	2–3.5 =3.6–4.5 = 4.6–5.5 = 5.6–6.11 = 7+ years of mental ages	Not found	/	/	/	/	Drumming with a stick	Hand/Forearm	Biological age: 13–23 years old
McCombe Waller and Whitall (2004)	Chronic hemiparesis vs. Healthy adults	No	• Paretic limb: Not found • Non-paretic limb: Chronic hemiparesis = Healthy adults	• Paretic limb: Not found • Non-paretic limb: Chronic hemiparesis = Healthy adults	Sensorimotor synchronization training in the non-paretic limb (in hemiparesis patients)	Pre faster than Post	Pre = Post sensorimotor synchronization training	Not found	Tapping on keys	Fingers	/
Martin et al. (2017)	Alzheimer's vs. Healthy elderly	No	Alzheimer's = Healthy elderly	Not found	/	/	/	/	Tapping on a keyboard key	Finger	/
Martínez Pueyo et al. (2016)	Huntington vs. Healthy adults	Yes	Huntington is slower than Healthy adults	Huntington is less stable than Healthy adults	/	/	/	/	Tapping on a keyboard key	Finger	/
Palmer et al. (2014)	2 Beat-deaf vs. Healthy adults	No	2 Beat-deaf = Healthy adults	2 Beat-deaf = Healthy adults	/	/	/	/	Tapping on a silent piano key	Finger	/
Phillips-Silver et al. (2011)	1 Beat-deaf (congenital amusia) vs. Healthy adults	Not found (case report)	Not found (case report)	Not found	/	/	/	/	Bouncing	Whole body	/
Provasi et al. (2014b)	Cerebellar medulloblastoma vs. Healthy children	Yes	Cerebellar medulloblastoma is slower than Healthy children	Cerebellar medulloblastoma is less stable than Healthy children	Sensorimotor synchronization task Sex	Pre faster than Post Male = Female	Pre = Post sensorimotor synchronization task Female = Male	• Interaction Pathology × Sensorimotor synchronization task: effect of Sensorimotor synchronization on SMT value and its stability is higher in Cerebellar medulloblastoma than in Healthy children. • No interaction Sex × Pathology × Sensorimotor synchronization task	Tapping on a keyboard key	Finger	/
Roche et al. (2011)	DCD vs. Healthy children	Yes	DCD = Healthy children	DCD is less stable than Healthy children	Sensory feedback	Vision+ Audition = No vision + Audition = Vision + No audition = No vision + No audition	Vision+ audition = No vision + Audition = Vision + No audition = No vision + No audition	No interaction Pathology × Sensory feedback	Anti-phase tapping on a table	Fingers	/
Roerdink et al. (2009)	Stroke vs. Healthy adults	Yes	Stroke is slower than Healthy adults	Not found	/	/	/	/	Walking on treadmill	Legs	/
Rose et al. (2020)	Parkinson's vs. Healthy elderly vs. Younger healthy adults	Yes (in all tasks)	• Finger tapping: Parkinson's = Healthy elderly// Parkinson's faster than Younger healthy adults// Healthy elderly (515 ms) faster than Younger healthy adults • Toe tapping: Parkinson's faster Healthy elderly = Younger healthy adults • Stepping: Parkinson's faster than Younger healthy adults// Parkinson's = Heatlthy elderly// Healthy elederly = Younger healthy adults	• Finger tapping: Parkinson's = Younger healthy adults// Parkinson's less stable than Healthy elderly// Younger healthy adults less stable than Healthy elderly • Toe tapping: Parkinson's = Younger healthy adults = Healthy elderly • Stepping: Parkinson's = Younger healthy adults = Healthy elderly	/	/	/	/	• Tapping on a stomp box • Tapping on a stomp box • Stepping on the spot	• Finger • Toe • Feet	
Rubia et al. (1999)	ADHD vs. Healthy children	Yes	ADHD = Healthy children	ADHD less stable than Healthy children	/	/	/	/	Tapping on a button	Finger	/
Schwartze et al. (2011)	Stroke (Basal ganglia lesions) vs. Healthy adults	Yes	Not found	Stroke less stable than Healthy adults	Sensorimotor synchronization task	Not found	Pre less stable than Post	No interaction Pathology × Sensorimotor synchronization task	Tapping on a copper plate	Hand	/
Schwartze et al. (2016)	Cerebellar lesion vs. Healthy adults	Yes	Cerebellar lesion = Healthy adults	Cerebellar lesion less stable than Healthy adults	Sensorimotor synchronization task	Pre = Post	Not found	No interaction Pathology × Sensorimotor synchronization task	Tapping on a pad	Finger	/
Schellekens et al. (1983)	Minor neurological dysfunction vs. Healthy children	Yes	Minor neurological dysfunction slower than Healthy children	Minor neurological dysfunction less stable than Healthy children	/	/	/	/	Pressing buttons	Hand/Arm	/
Volman et al. (2006)	DCD vs. Healthy children	Yes (in both tapping modes)	• In-phase: DCD slower than Healthy • Anti-phase: DCD slower than Healthy	Not found	Limb combination	• In-phase: Hand-foot ipsilateral = Hand-foot controlateral slower than Hand-hand • Anti-phase: Hand-foot ipsilateral = Hand-foot controlatéral slower than Hand-hand	• In-phase: Not found • Anti- phase: Not found	No interaction Pathology × Limb combination (for In-phase and Anti-phase)	In-phase and Anti-phase bi-effectors tapping on a pad	Hand and foot	Limb combinations: - Hand–hand coordination (homologous); - Hand–foot coordination same body side (ipsilateral) - Hand-foot coordination different body side (contralateral)
Wittmann et al. (2001)	Adults with Brain subcortical injury left hemisphere without aphasia (LHsub) vs. Brain cortical injury left hemisphere with aphasia (LH) vs. Brain cortical injury right hemisphere (RH) vs. Controls (orthopedic but not brain injury; CTrl)	Yes	LH slower than CTrl LHsub faster than CTrl RH = CTrl	LH = LHsub = RH = CTrl	Side (in controls)	Left = Right	/	/	Tapping on a keyboard key	Finger	/
Wurdeman et al. (2013)	Transtibial amputee vs. Healthy adults	No	Transtibial amputee = Healthy adults	Not found	/	/	/	/	Walking on a treadmill	Legs	/
Yahalom et al. (2004)	Parkinson's vs. Healthy elderly	No	Parkinson's = Healthy elderly	Parkinson's = Healthy elderly	/	/	/	/	Tapping on a board	Fingers	Subgroups of Parkinson's (Tremor predominant vs. Freezing predominant vs. Akinetic rigid vs. Unclassified) Freezing predominant Parkinson's vs. Unclassified Parkinson's adults significantly different
	**2. Age**	**Significance**	**Direction of the effect (on mean or median of SMT)**	**Direction of the effect (on the SD or Coefficient of variation of SMT)**	**Other factor(s)**	**Direction of the effect (on mean or median of SMT)**	**Direction of the effect (on the SD or Coefficient of variation of SMT)**	**Interaction**	**Task(s)**	**Effector(s)**	**Other information**
Baudouin et al. (2004)	21–35 vs. 66–80 vs. 81–94 years old	Yes	21–35 faster than 66–80 = 81–94 years old	Not found	/	/	/	/	Tapping on a plastic block	Finger	/
Drake et al. (2000)	4 vs. 6 vs. 8 vs. 10 years old children vs. Adults	Yes	Younger faster than Older	Younger more stable than Older	Trial measurement Musical expertise	Trial 1 slower than Trial 5 Non-musicians faster than Musicians	Not found Non-musicians less stable Musicians	No interaction Age × Trial measurement × Musical expertise	Drumming with a stick	Hand/forearm	/
Droit et al. (1996)	31–35 vs. 37–39 weeks of postmenstrual age in brain-damaged and low risks preterm infants	No	31–35 = 37–39 weeks of postmenstrual age	Not found	/	/	/	/	Kicking	Legs	/
Ejiri (1998)	Before vs. After onset of canonical babbling (CB)	Yes	Onset CB faster than Before and After CB	Not found	Audibility of rattles Weight of rattles Sex Side	Audible faster than Inaudible Not found Not found Not found	Not found Not found Not found Not found	Interaction Onset CB × Audibility of rattle: after onset CB, Audible rattle is faster than Inaudible.	Shaking a rattle	Arm	/
Fitzpatrick et al. (1996)	3 vs. 4 vs. 5 vs. 7 years old children	No	3 = 4 = 5 = 7 years old	Not found	Side Loading	Left = Right Not found	Not found Not found	Interaction Side × Loading: the right limb loaded oscillates faster than the left limb loaded.	Clapping with and without inertial loading limbs	Hands	/
Gabbard and Hart (1993)	4 vs. 5 vs. 6 years old children	Yes	Older faster than Younger	Not found	Sex Laterality	Male = Female Right = Mixed = Left	Not found Not found	No interaction Age × Sex × Laterality	Tapping on a pedal	Foot	/
Getchell (2006)	4 vs. 6 vs. 8 vs. 10 years old children vs. Adults	Yes	4 faster than 6 = 8 =10 years old = Adults	4 = 6 = 8 = 10 years old less stable than Adults	Dual task	Single faster than Dual	Dual less stable than Single	No interaction Age × Dual task	Striking cymbals	Hands/forearms	Other walking task (GAITRite)
Hammerschmidt et al. (2021)	7–49 years old	Yes	Younger faster than Older	Not found	Time of day Arousal Long-term stress Musical expertise	Earlier slower than Later Very calm = Rather calm = Neutral = Rather excited = Very excited Low stress = Moderate stress = High stress Non-musicians slower than Musicians	Not found Not found Not found Not found	Not found	Tapping on a keyboard key, or a mouse key, or a touchscreen of a tablet or a smartphone	Finger	Clusters analysis-based on SMT values
James et al. (2009)	6 vs. 10 years old children vs. Adults	Yes	6 years old faster than Adults	Younger less stable than Older	Support for rocking	Supported = Unsupported	Significant effect (no other information)	Interaction Age × Supported rocking on SMT and its stability: - When the feet were unsupported, only 6 year old were faster than Adults - Only 6 and 10 years old children are more stable with unsupported rocking.	Body rocking	Whole body	/
McAuley et al. (2006)	4–5 vs. 6–7 vs. 8–9 vs. 10–12 years old children vs. 18–38 vs. 39–59 vs. 60–74 vs. 75+ years old adults	Yes	Younger faster than Older	Not found	/	/	/	/	Tapping on a copper plate	Hand	Correlation analysis
Monier and Droit-Volet (2018)	3 vs. 5 vs. 8 years old children vs. Adults	Yes	• In non-emotional context: 3 = 5 = 8 years old faster than Adults • In emotional context: 3 = 5 = 8 years old faster than Adults	• In non-emotional context: 3 less stable than 5 less stable than 8 years old = Adults • In emotional context: 3 less stable than 5 less stable than 8 years old less stable than Adults	• Emotional context • Sex	• High-Arousal faster than Low-Arousal = Neutral • Male = Female	• High-Arousal more stable than Low-Arousal = Neutral • Male = Female	No interaction Age × Emotional context	Tapping on a keyboard key	Finger	/
Monier and Droit-Volet (2019)	5 vs. 6 vs. 7 years old children	Yes	5 = 6 = 7 years old	5 less stable than 6 less stable than 7 years old	Trial measurement	Trial 1 = Trial 2 = Trial 3	Trial 1 = Trial 2 = Trial 3	/	Tapping on a keyboard key	Finger	Linear regression analysis for age
Provasi and Bobin-Bègue (2003)	2½ vs. 4 years old children vs. Adults	Yes	Younger faster than Adults	Younger less stable than Older	Sensorimotor synchronization task	Pre faster than Post	Pre = Post	Not found	Tapping on a computer screen	Hand	/
Rocha et al. (2020)	4–37 months old infants	Yes	Younger slower than Older	Younger less stable than Older	/	/	/	/	Drumming	Hand	Correlation analysis
Vanneste et al. (2001)	24–29 years old adults vs. 60–76 years old elderly	Yes	24–29 faster than 60–76 years old	26 = 69 years old	Session measurement	Significant effect (no other information)	Session 1 = Session 2 = Session 3 = Session 4 = Session 5	Interaction Age × Session measurement: - Session 1 slower than Session 2 = Session 3 = Session 4 = Session 5 in Younger. - Session 1 slower than Session 2 slower than Session 3 = Session 4 = Session 5 in Oldest.	Tapping on a plastic block	Hand	/
Yu and Myowa (2021)	18 vs. 30 vs. 42 months old children	No	18 = 30 = 42 years old	Not found	/	/	/	/	Drumming with a stick	Hand/forearm	/
	**3. Effector/side**	**Significance**	**Direction of the effect (on mean or median of SMT)**	**Direction of the effect (on the SD or Coefficient of variation of SMT)**	**Other factor(s)**	**Direction of the effect (on mean or median of SMT)**	**Direction of the effect (on the SD or Coefficient of variation of SMT)**	**Interaction**	**Task(s)**	**Effector(s)**	**Other information**
Byblow and Goodman (1994)	Left vs. Right	No (in both coordination modes)	• Single rhythmic 1:1 coordination: Left = Right • Polyrhythmic 2:1 coordination: Left = Right	• Single rhythmic 1:1 coordination: Left = Right • Polyrhythmic 2:1 coordination: Left = Right	Session measurement	• Single rhythmic 1:1 coordination: Session 1 = Session 2 = Session 3 • Polyrhythmic 2:1 coordination: Not found	• Single rhythmic 1:1 coordination: Session 1 = Session 2 = Session 3 • Polyrhythmic 2:1 coordination: Not found	Not found (for single and polyrhythmic coordination)	• Single rhythmic 1:1 coordination • Polyrhythmic 2:1 coordination	• Forearm • Forearm	No comparison between the 2 modes of coordination
Getchell et al. (2001)	Right finger tapping in-phase; right finger tapping antiphase; arms clapping alone; lead leg galloping alone; lead leg galloping with clapping; arms clapping with galloping; right leg crawling	Tasks not compared	Not found (tasks not compared)	Not found (tasks not compared)	/	/	/	/	• Tapping on a key • Clapping • Galloping	• Finger • Arms • Legs	Correlation analyses between tasks
Kay et al. (1987)	Left vs. Right	No	• Single: Left = Right • Bimanual: Left = Right in Mirror and Parallel	• Single: Left = Right • Bimanual: Left = Right	• Mode of production • Session measurement	• Single = Mirror faster than Parallel • Session 1 = Session 2	• Single = Mirror = Parallel • Session 1 = Session 2	Not found	• Producing single flexion and extension • Producing bimanual flexion and extension	• Wrist • Wrist	/
Rose et al. (2021)	Finger vs. Foot vs. Whole body	No	Finger = Foot = Whole body	Not found	Age	Younger = Older	Not found	No interaction Effector × Age	• Tapping on a stomp box • Tapping on a stomp box • Stepping on the spot	• Finger • Foot • Whole body	/
Sakamoto et al. (2007)	Arm vs. Leg	Yes	Arms slower than Legs	Not found	/	/	/	/	• Pedaling • Pedaling	• Arms • Legs	/
Tomyta and Seki (2020)	1 Finger vs. 4 Fingers vs. Hand/Forearm	No	Not found	1 Finger = 4 Fingers = Hand/Forearm	/	/	/	/	• Tapping on (a) keyboard key(s) • Drumming with a stick	• Finger(s) • Hand/ Forearm	/
Whitall et al. (1999)	Left vs. Right	No	Left = Right	Not found	Mode of tapping	In-phase faster than Anti-phase	In-phase less stable than Anti-phase	Not found	Tapping on keyboard keys	Fingers	/
	**4. Expertise/predisposition**	**Significance**	**Direction of the effect (on mean or median of SMT)**	**Direction of the effect (on the SD or Coefficient of variation of SMT)**	**Other factor(s)**	**Direction of the effect (on mean or median of SMT)**	**Direction of the effect (on the SD or Coefficient of variation of SMT)**	**Interaction**	**Task(s)**	**Effector(s)**	**Other information**
Assaneo et al. (2021)	High vs. Low synchronization skill	Yes	High faster than Low	Not found	/	/	/	/	Producing a syllable	Mouth/lips	/
Bégel et al. (2022c)	Musicians vs. Non- musicians	Yes	Musicians = Non-musicians	Musicians more stable than Non-musicians	/	/	/	/	Tapping on a pad	Finger	/
Loehr and Palmer (2011)	Musicians vs. Non- musicians	No	Musicians = Non- musicians	Not found	/	/	/	/	Playing (one hand) a melody on a piano	Fingers	/
Scheurich et al. (2018)	Musicians vs. Non-musicians	Yes	Musicians slower than Non- musicians	Musicians more stable than Non- musicians	Trial measurement	Trial 1 slower than Trial 2 and Trial 3	Trial 1 = Trial 2 = Trial 3	No interaction Musical expertise × Trial measurement	Tapping a melody on one piano key	Finger	/
Scheurich et al. (2020)	Musicians vs. Non- musicians (experiment 2)	No	Musicians = Non-musicians	Not found	Trial measurement	Trial 1 slower than Trial 2 slower than Trial 3	Not found	No interaction Musical expertise × Trial measurement	Tapping on a force sensitive resistor	Finger	Percussionists excluded
Slater et al. (2018)	Musicians vs. Non- musicians	Yes	Not found	Musicians more stable than Non-musicians	/	/	/	/	Drumming	Hand	Percussionists
Tranchant et al. (2016)	High vs. Low synchronization skill	Yes	• Bouncing: High = Low synchronization skill • Clapping: High = Low synchronization skill	• Bouncing: High more stable than Low synchronization skill • Clapping: High = Low synchronization skill	Type of task	• In High synchronization skill: Clapping faster than Bouncing • In Low synchronization skill: Not found	• In High synchronization skill: Clapping more stable than Bouncing • In Low synchronization skill: Not found	/	• Bouncing • Clapping	• Whole body • Hands	/
	**5. Genotype**	**Significance**	**Direction of the effect (on mean or median of SMT)**	**Direction of the effect (on the SD or Coefficient of variation of SMT)**	**Other factor(s)**	**Direction of the effect (on mean or median of SMT)**	**Direction of the effect (on the SD or Coefficient of variation of SMT)**	**Interaction**	**Task(s)**	**Effector(s)**	**Other information**
Suzuki and Ando (2018)	Monozygotic vs. Dizygotic twins	No	Monozygotic = Dizygotic	Monozygotic = Dizygotic	Sex	Male = Female	Male = Female	Not found	Striking cymbals	Forearms/ Hands	Significant correlation between the tempo level of each Monozygotic twin but not between each Dizygotic twins
Wiener et al. (2011)	A1+ vs. A1- polymorphism Val/Val vs. Met+ polymorphism	• Yes • No	A1+ slower than A1 - Val/Val = Met+	A1+ = A1 - Val/Val = Met+	/	/	/	/	Tapping on a keyboard key	Not found	Subgroups of polymorphism [DRD2/ANKK1-Taq1a (A1–, A1+); COMT Val158Met (Val/Val, Met+); BDNF Val66Met (Val/Val, Met+)]
	**II. Intrinsic factors**										
	**1. Physical training**	**Significance**	**Direction of the effect (on mean or median of SMT)**	**Direction of the effect (on the SD or Coefficient of variation of SMT)**	**Other factor(s)**	**Direction of the effect (on mean or median of SMT)**	**Direction of the effect (on the SD or Coefficient of variation of SMT)**	**Interaction**	**Task(s)**	**Effector(s)**	**Other information**
Byblow et al. (1994)	Pre vs. Post sensorimotor synchronization	Yes	Pre slower than Post	Not found	Mode of coordination Side	Not found Not found	Not found Not found	Not found	Producing pronation and supination coordination	Forearms	/
Carson et al. (1999)	Pre vs. Post sensorimotor synchronization	Yes	Pre slower than Post	Pre = Post	Weighted coordination Side Mode of coordination	Heavy weight slower than No weight = Light weight Right slower than Left In-phase slower than Anti-phase	Heavy = No weight = Light weight Right = Left In-phase = Anti-phase	Not found	Coordinating flexing and extending elbow and wrist joints	Arm	/
Collyer et al. (1994)	Pre vs. Post sensorimotor synchronization	No	Pre = Post	Not found	Trial measurement Session	Pre: Trial 1 slower than Trial 2 = Trial 3 Post: Trial 1 slower than Trial 2 = Trial 3 Session 1 = Session 2 = Session 3 = Session 4 = Session 5 = Session 6 = Session 7 = Session 8	Not found Not found	Not found	Tapping on a plastic box	Finger	/
Dosseville et al. (2002)	Pre vs. Post physical exercise of pedaling	Yes	Pre slower than Post	Not found	Trial measurement Time of day	Pre: Trial 1 = Trial 2 = Trial 3 = Trial 4 Post: Trial 1 = Trial 3 6 pm faster than 6 am, 10 am and 10 pm//6 am slower than 2 pm	Not found Not found	Not found	Tapping on a table	Finger	/
Hansen et al. (2021)	Cadence of physical training: 50 rpm vs. 90 rpm vs. Freely chosen	Yes	50 rpm slower than Freely chosen 90 rpm faster than Freely chosen	Not found	/	/	/	/	Pedaling	Legs	/
Robles-García et al. (2016)	Pre vs. Post vs. 2 weeks Post imitation and motor practice vs. Motor practice alone in elderly with Parkinson's disease	No	Pre = Post = 2 weeks Post	Pre = Post = 2 weeks Post	Type of physical training Laterality	Imitation and motor practice = Motor practice alone Not found	Imitation and motor practice = Motor practice alone In Pre physical training: Dominant more stable than Non-dominant hand	No interaction Training × Type of physical training	Tapping	Finger	/
Rocha et al. (2021)	Pre vs. Post passive walking in non-walking infants	Yes	Pre = Post	Not found	Passive walking frequency	Fast = Slow	Not found	Interaction Training × Passive walking frequency: - Infant SMT in the Fast walking frequency became faster from pre to post training. - Infant SMT in the Slow condition became slower from pre to post training.	Drumming	Hands	/
Sardroodian et al. (2014)	Pre vs. Post 4 weeks of heavy strength training	No	Pre = Post	Not found	/	/	/	/	Pedaling	Legs	/
Turgeon and Wing (2012)	Pre vs. Post sensorimotor synchronization and continuation	No	Pre = Post	Pre = Post	Age	Younger faster than Older	Younger more stable than Older	Not found	Tapping on a mouse key	Finger	Linear regression analysis for age
Zamm et al. (2018)	Pre vs. Post faster or slower sensorimotor synchronization	No	Pre = Post	Not found	Time of day Age Sex	Earlier = Later Younger = Older Not found	Not found Not found Not found	Not found Not found Not found	Playing a melody on a piano	Fingers	Pianists Correlation analysis for age
Bouvet et al. (2019)	Ascending vs. Descending rhythmic stimuli (listening while trying not to synchronize) vs. Without rhythmic stimuli	Yes	Ascending faster than Descending rhythmic stimuli and Without rhythmic stimuli	Ascending stimulus less stable than Descending and Without rhythmic stimuli	Time of testing	Significant effect (no other information)	Significant effect (no other information)	Interaction Value modulation of stimuli time intervals × Time of testing: - Ascending more stable than Without rhythmic stimuli at the beginning of testing. - Ascending and Descending more stable than Without rhythmic stimuli at the end of testing.	Air tapping task (flexion and extension)	Finger	/
	**2. External constraints**	**Significance**	**Direction of the effect (on mean or median of SMT)**	**Direction of the effect (on the SD or Coefficient of variation of SMT)**	**Other factor(s)**	**Direction of the effect (on mean or median of SMT)**	**Direction of the effect (on the SD or Coefficient of variation of SMT)**	**Interaction**	**Task(s)**	**Effector(s)**	**Other information**
Bouvet et al. (2020)	One vs. Two vs. Three times the spontaneous motor tempo value as time intervals between stimuli (listening while trying not to synchronize)	Yes	One faster than Two and Three times the spontaneous motor value	One = Two = Three times the spontaneous motor value	Accentuation pattern Session Trial measurement	Unaccented = Binary accented = Ternary accented Session 1 = Session 2 Trial 1 = Trial 2 = Trial 3	Unaccented = Binary accented = Ternary accented Session 1 = Session 2 Trial 1 = Trial 2 = Trial 3	• No interaction Value of stimuli time intervals × Accentuation pattern • No interaction Session × Trial measurement	Air tapping task (flexion and extension)	Finger	/
Hansen and Ohnstad (2008)	200 m real vs. 3,000 m simulated altitude with loading on the cardiopulmonary system (experiment 1)	No	173 W at 200 m real = 173 W at 3,000 m simulated = 224 W at 200 m real	Not found	/	/	/	/	Pedaling	Legs	/
Hatsopoulos and Warren (1996)	0 kg vs. 2.27 kg vs. 4.55 kg external added mass	Yes	0 kg faster than 2.27 kg faster than 4.55 kg	Not found	Session External spring stiffness	Session 1 = Session 2 0 N/m slower than 47.34 N/m slower than 94.68 N/m slower than 142.02 N/m	Not found Not found	Interaction External added mass × External spring stiffness (no more information)	Arms swinging	Arms	/
Sofianidis et al. (2012)	No contact vs. Fingertip contact	Yes	No contact slower than Fingertip contact	Not found	Dance expertise	Expert dancers = Novice dancers	Not found	No interaction Contact interaction × Dance expertise	Body rocking	Whole body	/
Verzini de Romera (1989)	Quiet vs. Noisy environment	Yes	Noisy environment faster than Quiet	Not found	/	/	/	/	Not found	Not found	/
Wagener and Colebatch (1997)	0.35 Nm vs. 0.18 Nm vs. 0.26 Nm extension vs. 0.09 Nm vs. 0.18 Nm flexion torque load vs. without external load	No	0.35 Nm = 0.18 Nm = 0.26 Nm extension = 0.09 Nm = 0.18 Nm flexion = Without external load	Not found	/	/	/	/	Flexion and extension	Wrist	/
	**3. Observation training**	**Significance**	**Direction of the effect (on mean or median of SMT)**	**Direction of the effect (on the SD or Coefficient of variation of SMT)**	**Other factor(s)**	**Direction of the effect (on mean or median of SMT)**	**Direction of the effect (on the SD or Coefficient of variation of SMT)**	**Interaction**	**Task(s)**	**Effector(s)**	**Other information**
Aridan and Mukamel (2016)	Pre vs. Post passive observation of a rhythmic action	Yes	Pre slower than Post (only in subjects with “slower” spontaneous motor tempo at Pre training)	Not found	/	/	/	/	Tapping on keys	Fingers	Subgroups of spontaneous motor tempo profile in Pre training: Slow (slowest spontaneous motor tempo) vs. Fast (fastest spontaneous motor tempo)
Avanzino et al. (2015)	Pre vs. Post passive observation combined with Transcranial Magnetic Stimulation	Not found	Not found	Not found	Type of observation training (Passive observation of a rhythmic action vs. Passive observation of a landscape)	Not found	Not found	Interaction Passive observation training × Type of observation: Pre slower than Post only for Passive observation of a rhythmic action.	Performing an opposition sequence	Fingers	/
Bisio et al. (2015)	Pre vs. Post passive observation of a rhythmic action	Not found	Not found	Not found	Type of observation training (Passive observation of a rhythmic action vs. Passive observation of a rhythmic action combined with peripherical nerve stimulation vs. Peripherical nerve stimulation vs. Passive observation of a landscape)	Not found	Not found	Interaction Passive observation training × Type of observation: Pre slower than Post only for Passive observation of a rhythmic action combined with peripherical nerve stimulation.	Performing an opposition sequence	Fingers	/
Bove et al. (2009)	Pre vs. Post passive observation of a rhythmic action (after 45 min and 2 days)	No	Pre = Post 45 min = Post 2 days	Not found	Instruction Type of passive observation	Not found Not found	Not found 1 Hz more stable than 3 Hz rhythmic action and Landscape	• Interaction Type of Passive observation × Instruction: With instruction faster than without instruction only for passive observation of a 3 Hz rhythmic action. • Interaction Pre-Post × Type of observation: Pre less stable than Post only for passive observation of a 3 Hz rhythmic action	Performing an opposition sequence	Fingers	/
Lagravinese et al. (2017)	Type of passive observation: Passive observation of a rhythmic action vs. Passive observation of a metronome	Not found	Not found	Not found	Session	In Pre training: Session 1 slower than Session 2 slower than Session 3 = Session 4 = Session 5	Significant effect (no other information)	Interaction Type of passive observation × Session: - Day 5 faster than Day 1 only for Passive observation of a rhythmic action. - Day 5 more stable than Day 1 only for Passive observation of a metronome.	Performing an opposition sequence	Fingers	/
	**4. Time of testing**	**Significance**	**Direction of the effect (on mean or median of SMT)**	**Direction of the effect (on the SD or Coefficient of variation of SMT)**	**Other factor(s)**	**Direction of the effect (on mean or median of SMT)**	**Direction of the effect (on the SD or Coefficient of variation of SMT)**	**Interaction**	**Task(s)**	**Effector(s)**	**Other information**
Hansen and Ohnstad (2008)	Week 1 from Week 12 (experiment 2)	No	• Pedaling: No change across Weeks • Tapping: No change across Weeks	• Pedaling: Not found • Tapping: Not found	/	/	/	/	• Pedaling • Tapping on a pad	• Legs • Finger	/
Moussay et al. (2002)	6 am vs. 10 am vs. 2 pm vs. 6 pm vs. 10 pm	Yes	• Tapping: 6 am slower than 6 pm//6 pm faster than 10 pm • Pedaling: 6 am slower than 10 am, 2 pm, 6 pm, and 10 pm	• Tapping: Not found • Pedaling: Not found	/	/	/	/	• Tapping on a table • Pedaling	• Finger • Legs	Cyclists
Oléron et al. (1970)	Wake-up vs. Morning vs. Midday vs. Early afternoon after nap vs. Middle afternoon vs. Evening vs. Bed time	Yes	Wake-up slower than Morning	Not found	Staying in a cave	Beginning of staying in a cave slower than Ending of staying in a cave (linked to circadian rhythm modification)	Not found	Not found	Tapping on a Morse key	Finger	Significant effect only reported between Wake up and Morning
Schwartze and Kotz (2015)	Time 1 (Target) vs. Time 2 (Control)	Yes	Time 1 (Target) = Time 2 (Control)	Time 1 (Target) more stable than Time 2 (Control)	Age	Younger = Older	Younger = Older	Not found	Tapping on a pad	Finger	Correlation analysis for age
Wright and Palmer (2020)	9 am vs. 1 pm vs. 5 pm vs. 9 pm	Yes	9 am slower than 1 pm, 5 pm and 9 pm//1 pm slower than 9 pm	9 am less stable than 5 pm and 9 pm//1 am less stable than 9 pm	Familiar melody	Familiar slower than Unfamiliar	Familiar more stable than Unfamiliar	No interaction Time of testing × Familial melody	Playing (one hand) a melody on a piano	Fingers	Pianists
	**5. Internal state**	**Significance**	**Direction of the effect (on mean or median of SMT)**	**Direction of the effect (on the SD or Coefficient of variation of SMT)**	**Other factor(s)**	**Direction of the effect (on mean or median of SMT)**	**Direction of the effect (on the SD or Coefficient of variation of SMT)**	**Interaction**	**Task(s)**	**Effector(s)**	**Other information**
Boulanger et al. (2020)	Increasing vs. Decreasing gravity	Yes (but descriptive data)	Larger linear relationship with gravity in Increasing gravity than in Decreasing gravity (higher energetic cost in high gravity for a given change in frequency)	Not found	Session	Session 1 = Session 2	Not found	Not found	Performing upper arm movements	Arm	Mathematical data representing spontaneous motor tempo
Dosseville and LaRue (2002)	Apnea vs. No apnea	Yes	Apnea slower than No apnea	Not found	/	/	/	/	Tapping on a metal plate	Finger	/
Murata et al. (1999)	Mental stress vs. No mental stress	Yes	Mental stress faster than No mental stress	Mental stress less stable than No mental stress	Trial measurement (3 Trials with Mental stress)	Not found	Not found	Not found	Tapping a key	Finger	/
	**6. Type of task**	**Significance**	**Direction of the effect (on mean or median of SMT)**	**Direction of the effect (on the SD or Coefficient of variation of SMT)**	**Other factor(s)**	**Direction of the effect (on mean or median of SMT)**	**Direction of the effect (on the SD or Coefficient of variation of SMT)**	**Interaction**	**Task(s)**	**Effector(s)**	**Other information**
Forrester and Whitall (2000)	In-phase vs. Anti-phase	Yes	In-phase faster than Anti-phase	In-phase = Anti-phase	Fingers pairing	Index only slower than Middle only	Index only = Middle only = Index + Middle	No interaction Type of task × Fingers pairing	Bimanual tapping on keys	Fingers	/
Pfordresher et al. (2021)	Finger tapping vs. Playing a melody vs. Reciting a sentence (experiment 1)	Yes	Finger tapping slower than Playing a melody slower than Reciting a sentence (experiment 1)	Reciting a sentence more stable than Playing a melody and Finger tapping (experiment 1)	/	/	/	/	• Playing (one hand) a melody on a piano • Tapping on a piano key • Reciting a sentence	• Fingers • Finger • Mouth/lips	Correlations analyses on consistency across trials
	Playing a melody vs. Reciting a sentence (experiment 2)	Yes	Playing a melody slower than Reciting a sentence (experiment 2)	Reciting a sentence more stable than Playing a melody (experiment 2)							
Scheurich et al. (2018)	Tapping a melody vs. Playing a melody (experiment 1)	No	Tapping a melody = Playing a melody	Not found	Trial measurement	Trial 1 slower than Trial 2 slower than Trial 3	Not found	No interaction Type of task × Trial measurement	• Tapping a melody on one piano key • Playing (one hand) a melody on a piano	• Finger • Fingers	Correlations analyses on consistency across melodies
Tajima and Choshi (1999)	Polyrhythmic vs. Single rhythmic task	Yes	• Left hand: Polyrhythmic slower than Single rhythmic task (Trial 1, 2 and 3) • Right hand: Polyrhythmic slower than Single rhythmic task (Trial 1 and 2)	• Left hand: Polyrhythmic less stable than Single rhythmic task (Trial 1 and 2) • Right hand: Polyrhythmic less stable slower than Single rhythmic task (Trial 1, 2 and 3)	Sex	Male = Female	Male = Female	Not found	Tapping on Morse keys	Fingers	Differences reported separately for the right and the left hands and across trials
Zelaznik et al. (2000)	Tapping vs. Drawing	Yes	Tapping faster than Drawing	Drawing more stable than Tapping	/	/	/	/	• Tapping on a desk • Drawing a circle on a paper	• Finger • Fingers/Wrist	/
	**7. Dual task**	**Significance**	**Direction of the effect (on mean or median of SMT)**	**Direction of the effect (on the SD or Coefficient of variation of SMT)**	**Other factor(s)**	**Direction of the effect (on mean or median of SMT)**	**Direction of the effect (on the SD or Coefficient of variation of SMT)**	**Interaction**	**Task(s)**	**Effector(s)**	**Other information**
Aubin et al. (2021)	Selective vs. Divided vs. Sustained attentional conditions	No	Selective = Divided = Sustained	Selective = Divided = Sustained	/	/	/	/	Legs swinging	Legs	Dual task
Serrien (2009)	Single motor task vs. Dual motor and verbal counting task	Not found	Not found	Not found	Side (Left vs. Right vs. Bimanual)	Not found	Not found	Interaction Dual task × Side: In Bimanual mode, Dual slower than Single	Tapping on a keyboard	Finger(s)	/

Factors are classified as intrinsic and extrinsic. Significance is reported as YES if one of the dependent variables (mean, median, standard deviation, or coefficient of variation of SMT) is significantly different between modalities of the main factor studied. The effectors used to perform the task are reported. Other information is reported if mentioned in the studies, particularly the effects of other secondary factors or interactions. The directions of effects of the main and other factors on the dependent variable(s) are reported. The directions of effects are reported as Not found when no statistics were performed on the dependent variable, when the dependent variable was not studied, or when the direction of the effect or the interaction was not explicitly reported.

**Figure 2:**
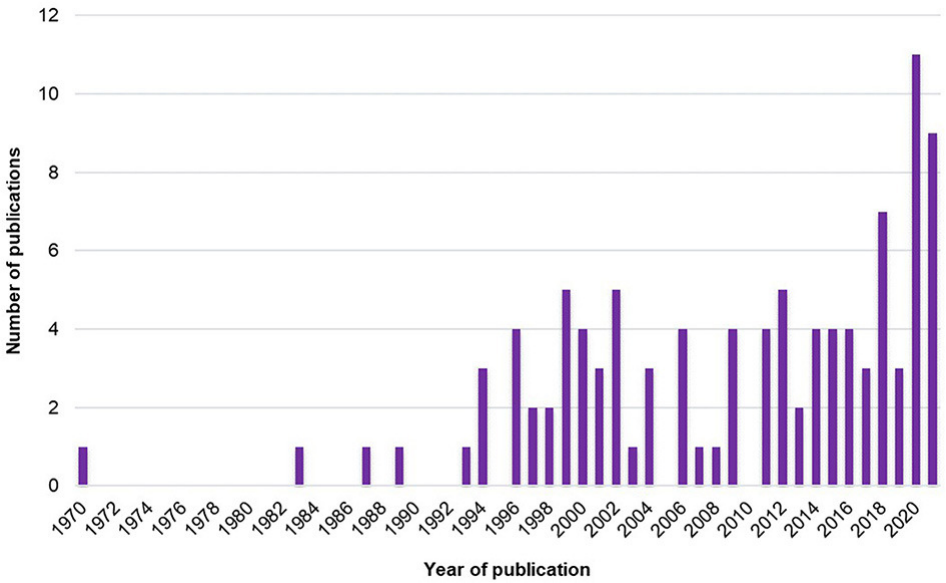
Number of studies exploring the SMT across years.

The number of studies exploring the SMT across years is presented in Figure 2.

## 4. Discussion

The present systematic review aimed to (1) characterize the range of SMT values found in the literature in healthy human adults and (2) identify all the factors modulating the SMT values in humans.

First, it is interesting to note that the global number of studies has grown since the early 1970's (Figure 2). The increase in studies about SMT actually started in the mid-1990's and has grown non-linearly to reach a peak in 2020. Thus, interest in SMT is old but has recently increased.

Second, our results highlight that (1) the reference value of SMT is far from being a common value of 600 ms in healthy human adults, but a range of SMT values exists and (2) many factors modulate the SMT values. We discuss these factors according to a classification as *intrinsic factors*, in relation to personal characteristics, and *extrinsic factors*, in relation to environmental characteristics. We also provide recommendations to measure, report, and use the SMT values for future studies on rhythmic production and perception.

### 4.1. Range of SMT values in healthy human adults

Regarding the range of SMT values, we have selected the studies that propose an SMT task as a baseline, followed by a second task that is usually a sensorimotor synchronization task, without comparison between factors or conditions (Table 3). However, no value of SMT is reported in some studies (*N* = 2/13). Hence, it is important to measure the SMT as a baseline before any rhythmic task and to report the SMT values in order to interpret the results with regard to this baseline.

The number of studies measuring the SMT as a baseline for a rhythmic task (to adjust the tempo of the rhythmic task) is rather low (Table 3), compared to those testing the effects of variables on the SMT values (Table 4). This may be due to the fact that the terminology used to designate the spontaneous motor tempo is heterogeneous. Although the SMT was clearly defined by Fraisse (1974) as the speed that the subject considers most natural and pleasant (p. 50), this terminology is not unanimous. Although some authors use the term “spontaneous motor tempo” (Drake et al., 2000; McPherson et al., 2018; Amrani and Golumbic, 2020), others use different terms, such as “preferred motor tempo” (Michaelis et al., 2014), “preferred rate” (McCombe Waller and Whitall, 2004), “preferred frequency” (Volman et al., 2006; Bouvet et al., 2020), “internal clock” (Yahalom et al., 2004), “spontaneous production rate” (Wright and Palmer, 2020), “motor spontaneous tempo” (Dosseville and LaRue, 2002; Moussay et al., 2002), “spontaneous movement tempo” (Avanzino et al., 2015; Bisio et al., 2015), “freely chosen cadence” (Sidhu and Lauber, 2020; Hansen et al., 2021), or “personal tempo” (Tajima and Choshi, 1999). In the same vein, the term “self-paced” is not used with a consensual definition. Sometimes, this term relates to an intentional spontaneous motor behavior without a rhythmic component, even if authors use the term “self-paced tapping” (e.g., Bichsel et al., 2018, not included in the present review), and sometimes it relates to an intentional spontaneous rhythmic motor behavior when “self-paced” is followed by “tempo” (Serrien, 2009; Hattori et al., 2015). For future studies measuring the SMT, we recommend using the terminology “spontaneous motor tempo” when the participant is invited to produce a rhythmic motor task not induced by external stimuli specifying a required tempo. The term “spontaneous motor tempo” should be preferred to the term “self-paced” to define the task. To increase the visibility of studies implying SMT, the term “spontaneous motor tempo” and its acronym “SMT” should appear in the title or keywords of the articles.

The tasks used to measure the SMT are also very heterogeneous. Even if Fraisse (1974) declared that SMT is commonly measured during a manual task (Fraisse, 1974), our results reveal that studies exploring SMT also measure other effectors apart from manual ones. Some studies use self-paced tapping with one or two effectors; others use drumming, hopping, pointing, cycling, swaying, and producing syllables; and another uses jaw opening-closing and chewing (Table 3). Regarding the SMT values, participants seem to be slower when the whole body or the jaw is required, compared to manual responses. Thus, the heterogeneity of effectors (finger, arm, leg, whole body, mouth/lips, and jaw) used to produce the SMT could explain the heterogeneity of results. This hypothesis could be in accordance with the results of Sakamoto et al. (2007), highlighting that the SMT is effector-dependent (Sakamoto et al., 2007), but we recommend to carry out further studies to test the impact of effectors on SMT.

The range of SMT values (from 333 to 3,160 ms) is far from being a common value of 600 ms, as first reported by Fraisse (1974). More specifically, it is important to note that studies reporting the slowest SMT values involve cyclical movements compared to the discrete isochronous movements of tapping or clapping. Regarding finger tapping, SMT appears to be faster (from 333 to 931 ms). Bouvet et al. (2020), who investigate the effect of accents and subdivisions in synchronization, performed a measurement of SMT during finger-tapping with a large number of taps in several trials. They also find a faster value around 650 ms. The heterogeneity of results can be explained by the heterogeneity in the paradigm applied to measure the SMT in the studies. We provide such examples in the following paragraphs.

First, the characteristics of participants are not homogeneously reported, particularly their level of musical experience. In some studies listed in Table 3, authors report that participants have no musical training. Note that some studies mix musicians and non-musicians in their samples (e.g., Michaelis et al., 2014; De Pretto et al., 2018). However, three studies reported in Table 4 show an effect of music expertise (Drake et al., 2000; Slater et al., 2018; Hammerschmidt et al., 2021). Information about musical expertise is particularly important, including the expertise of listening to music, given that it is possible that participants could present amusia or a deficit in rhythm production or perception (Stewart et al., 2006; Clark et al., 2015; Peretz, 2016; Sarasso et al., 2022). To have a better overview of the range of SMT values in healthy adults without musical expertise, we recommend reporting a general level of musical experience, that is, both the level of expertise in music/rhythm production and music/rhythm exposure.

Second, the characteristics of participants are also heterogeneous across studies in terms of age, sex, and laterality. Regarding the age, participants are from 18 to 45 years old (Table 1). Despite the fact that the age range is representative of healthy young adults, the range of SMT values varies in five studies about manual responses from 333 to 1,100 ms (Michaelis et al., 2014; De Pretto et al., 2018; McPherson et al., 2018; Zhao et al., 2020). Regarding the sex repartition, only two studies recruit an equal number of women and men (Michaelis et al., 2014; De Pretto et al., 2018); the others recruit either more women or more men. As reported in Table 4, the effect of sex on SMT has not been extensively studied, given that only one study addresses this question and reports no significant results (Suzuki and Ando, 2018). Regarding the laterality, the majority of studies do not report the laterality of participants (Table 3, *N* = 8/13). The other studies generally recruit right-handed participants (Table 3, *N* = 3/5). Some studies include one or two left-handed participants (Table 3, *N* = 2/5). In Table 4, no study investigates the effect of laterality on the SMT values. In the absence of clear results about laterality, we recommend specifying the laterality of the participants by means of a laterality questionnaire (e.g., Oldfield, 1971) in the case of a SMT task performed with a lateralized effector (hand or leg). More globally, to have a better overview of the range of SMT values in healthy adults, we recommend reporting the age, sex, and laterality of participants and specifying, if possible, whether the SMT differs according to these variables.

Third, how the SMT is *measured* is not consistent across studies (Table 3). As specified in Table 3, SMT paradigms differ according to the number of trials and their duration, as well as to the instructions provided to the participants. The number and duration of trials vary across studies. Globally, the number of trials is from 1 to 10, and the duration of each trial can be expressed as a range of time (seconds or minutes), a number of responses, or a number of inter-response intervals (Table 3). Two studies do not report any information about trials (Ruspantini et al., 2012; Malcolm et al., 2018). Regarding the instructions, it is important to note that the instructions are *not* reported in three out of 13 studies (Eriksson et al., 2000; Hattori et al., 2015; Malcolm et al., 2018). When reported, the instructions contain the terms “natural,” “comfortable,” “most comfortable,” “naturally comfortable,” “preferred,” “steady,” “freely chosen,” “own self-selected,” “spontaneously,” “without mental effort,” “do not require much awareness,” “without fatigue,” and “could be performed all day if necessary,” to characterize the manner to produce the SMT (Table 3). Moreover, the tempo itself is characterized as “tempo,” “pace,” “cadence,” “speed,” “rate,” and “frequency.” Even if these terms are supposed to represent the same instruction, we would like to emphasize that the semantics is not a detail. The instruction can modify the participant's behavior depending on the interpretation he/she makes of it. For example, the term “speed” can be interpreted by participants as an instruction to go fast. Thus, to have a better overview of the range of SMT values in healthy adults, we recommend reporting exactly and exhaustively the standardized instructions given to participants. More precisely, we recommend giving priority to the notions of “preferred,” “spontaneous,” and “comfortable tempo,” in the instructions given to the participant. It seems important to avoid the notion of “speed” in order not to induce the idea of performing the task as quickly as possible.

Fourth, how SMT is *recorded and computed* is not consistent. Regarding the measurement recordings, authors report the inter-response interval, frequency, number of movement cycles during the total duration of the trial, rate, cycle time, speed, or cadence. If reported, the values also have different units (milliseconds, seconds, beats per minute, Hertz, repetitions per minute, or kilometers per hour). Furthermore, the authors usually report the range of SMT values, the SMT mean and/or median, its standard deviation, and/or the coefficient of variation (Table 3). These discrepancies are probably due to the type of task used. Only two studies recording SMT do not report any value for SMT (LaGasse, 2013; Zhao et al., 2017). On this basis, we recommend reporting the SMT values when recorded and homogenizing the measurement recording, the variables, and their units (in milliseconds or Hz). It is, therefore, necessary to report, at least, the SMT values with the median and the range of SMT values with a box plot representing individual values to get access to the distribution of data with the minimal and maximal values. It is also important to specify the methodology to compute the SMT, in particular to report excluded data, for example, the first responses that were performed by the participants, which can be considered warm-up.

### 4.2. Intrinsic and extrinsic factors modulating SMT values

**Figure 3:**
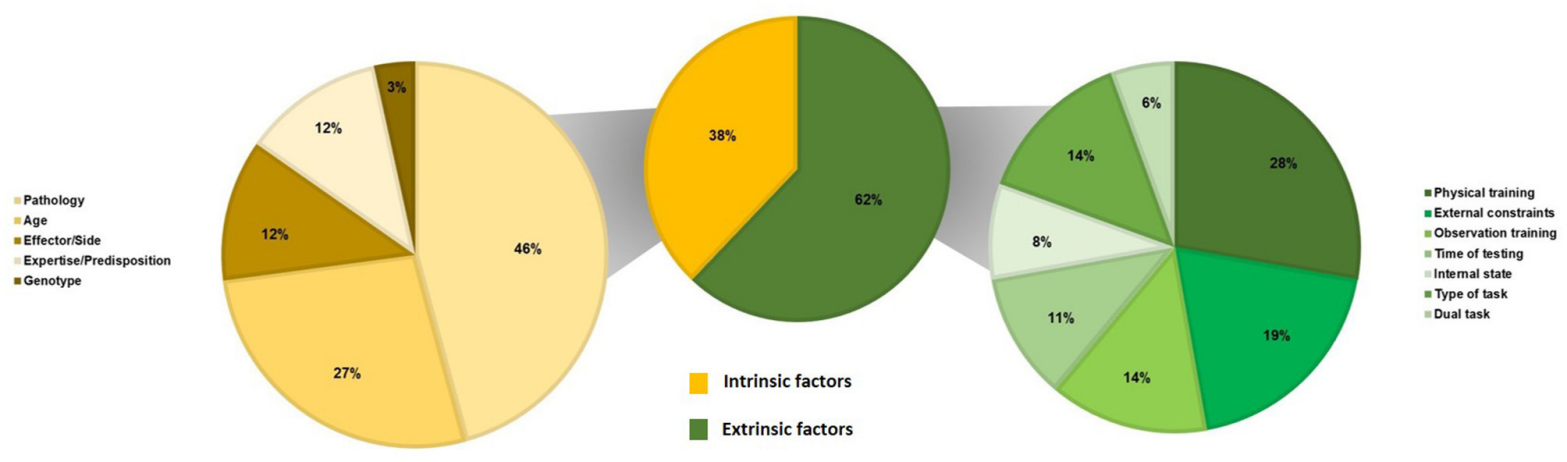
Repartition of studies on the factors modulating the SMT values (*N* = 94) according to intrinsic factors (*N* = 59) and extrinsic factors (*N* = 36).

Table 4 summarizes the results of studies about factors that could modulate the SMT values. We classified these factors as intrinsic and extrinsic ones, i.e., factors that could explain inter- and intra-individual variability in SMT values. Figure 3 presents the repartition of studies about the factors modulating the SMT values according to the intrinsic factors (*N* = 59) and the extrinsic factors (*N* = 36).

Regarding the intrinsic factors, our results reveal that the SMT is affected by several factors such as pathology, age, effector, expertise, or genotype (see Table 4). First, our results reveal that several pathologies modify the SMT values. Studies investigate brain lesions (six on Parkinson's, four on stroke, one on Huntington disease, one on Alzheimer's disease, one on Whiplash, and two on cerebellar lesions), neurodevelopmental disorders (two on attention deficit and hyperactivity disorder, two on developmental coordination disorder, one on developmental intellectual deficit, one on stuttering, and one on minor neurological dysfunction), and mental disorders (two on schizophrenia). Two studies test the effects of a deficit in music perception (beat deafness, i.e., difficulties in tracking or moving to a beat), and only one study examines the effect of an amputation. Globally, our results show that the most studied pathologies are brain lesions. Results indicate quasi-unanimously that SMT is affected by brain lesions (Table 4, *N* = 12/15). Studies report that either the frequency or the stability of the SMT differs in brain-injured patients compared to controls. In brain lesions, neurodegenerative disorders are the most studied, such as Parkinson's and Huntington's diseases (both implying a lesion of the basal ganglia) or Alzheimer's disease. Studies on Parkinson's disease report quasi-consistently that SMT is significantly affected in patients compared to healthy elderly individuals (Table 4, *N* = 5/6), and the study on Huntington's disease reports the same effect (Martínez Pueyo et al., 2016). The only study on Alzheimer's disease does not report any difference between patients and healthy elderly individuals (Martin et al., 2017). Moreover, most of the studies report that SMT is significantly affected in patients with stroke compared to healthy adults (Table 4, *N* = 3/4). In contrast, results are less consistent for neurodevelopmental and mental disorders. Attention deficit and hyperactivity disorder seems to affect the SMT (Table 4, *N* = 2/2), as does developmental coordination disorder (Table 4, *N* = 2/2). Only two studies report the effects of beat deafness with no consistent results (Phillips-Silver et al., 2011; Palmer et al., 2014). Based on these results, it is interesting to note that the SMT is affected regardless of the location of the lesion (motor cortex, language areas, basal ganglia, or cerebellum) and regardless of the physiopathology (neurodegenerative vs. neurological vs. neurodevelopmental). Although it seems more likely that focal lesions affect the SMT, future studies are required to better understand if and how the SMT is affected by neurodevelopmental, mental, and sensory disorders.

A second factor modulating the SMT is age. Studies investigate mostly infants (Table 4, *N* = 14/16). Only three studies investigate the elderly (Vanneste et al., 2001; Baudouin et al., 2004; McAuley et al., 2006). Our results reveal that age modifies the value of the SMT in the majority of studies (Table 4, *N* = 11/14). In fact, only three out of 14 studies do not find an effect of age in infants or children (Droit et al., 1996; Fitzpatrick et al., 1996; Yu and Myowa, 2021). It is interesting to note that only two studies test the possible effects of age on the SMT in individuals between 18 and 60 years old (McAuley et al., 2006; Hammerschmidt et al., 2021). Anyway, our results suggest that future studies about the SMT should take into account the effect of age bands or include the age of participants as a covariate, especially if participants are infants or elderly individuals.

A third intrinsic factor modulating the SMT is the effector/side used to produce the task. Results are very contradictory, with one study revealing an effect of the effector (Sakamoto et al., 2007) and two studies failing to reveal this effect (Tomyta and Seki, 2020; Rose et al., 2021). It seems that there is no effect of the side of the hand producing the SMT (Kay et al., 1987; Byblow and Goodman, 1994; Whitall et al., 1999). Moreover, it is also possible that SMT differs when it is produced with arms and legs (Sakamoto et al., 2007). Finally, the study of Getchell et al. (2001) reveals a correlation between SMT produced by different effectors. This result suggests that individuals have a general ability to produce their own SMT regardless of the type and number of effectors used (Getchell et al., 2001). Given that only one study reports this finding, further studies are required to confirm this effect.

As previously discussed above, expertise in music seems to modify the SMT. Musicians seem to have a more stable SMT than non-musicians (Scheurich et al., 2018; Slater et al., 2018; Bégel et al., 2022c). Moreover, two studies suggest that a predisposition to high or low synchronization (i.e., good or poor synchronization skills in rhythmic synchronization tasks) alters the SMT (Tranchant et al., 2016; Assaneo et al., 2021). Even if long-lasting intensive training could modify the SMT in certain conditions, it seems that intrinsic predispositions could be important. This result is in accordance with the last intrinsic factor identified in the current literature review, namely, the genotype. Two studies focus on this factor (Wiener et al., 2011; Suzuki and Ando, 2018). The first study finds a significant correlation between the tempo level in monozygotic twins but not in dizygotic twins, thereby suggesting that the genetic code could have a role in the SMT values (Suzuki and Ando, 2018). However, no difference between women and men is found, thereby preventing the possible role of sex on the SMT values (Suzuki and Ando, 2018). The second study reveals a significant effect of a polymorphism (Wiener et al., 2011). If we consider that one polymorphism (A1+) seems implied in the regulation of the density of receptors in the striatum (see Wiener et al., 2011), this result is in accordance with the results of studies showing an effect of Parkinson's disease, which affects the striatum, on the SMT (Konczak et al., 1997; Byblow et al., 2002; Flasskamp et al., 2012; Rose et al., 2020; Horin et al., 2021). Even if further studies are required to confirm this hypothesis, there is evidence that the genotype plays a role in the SMT values.

Regarding the extrinsic factors, our results highlight that the SMT is affected by several factors such as physical training, external constraints, observation training, time of testing, type of task, or dual tasking (see Table 4).

In total, 10 studies report results about the effects of physical training on the SMT. Six studies reveal a significant effect of cycling, strength training, synchronization, or physical exercise on the SMT values measured before and after training (Table 4). This result suggests that all studies about SMT should report the activity preceding the measurement of the SMT, especially physical activity.

In the same vein, all the studies (*N* = 5) testing the effects of the observation of a rhythmic action on the SMT found a significant effect (see Table 4). This result indicates that observing a rhythmic action without moving or synchronizing with it induces a spontaneous change in the SMT. This result is in accordance with the results of studies about the effects of physical training with rhythmic stimuli (Byblow et al., 1994; Carson et al., 1999; Hansen et al., 2021; Rocha et al., 2021). They are also in accordance with results about the effect of external constraints that show a significant effect of producing SMT while listening to a rhythmic metronome without synchronizing (Bouvet et al., 2019). The effect of observation or listening could be related to the implication of the Mirror System that is activated during observation, listening, and action (Kohler et al., 2002; Rizzolatti and Craighero, 2004). More precisely, it is possible that observing/listening a rhythm activates the same cerebral areas (i.e., the fronto-parietal system) as synchronizing to rhythmic stimuli (Konoike and Nakamura, 2020), hence modifying the SMT values according to the observed/listened tempo.

Regarding the effect of a dual task on the SMT, only one of the two studies reports a significant difference in the SMT during a single vs. dual task (Serrien, 2009). In the other study (Aubin et al., 2021), participants were instructed to swing their legs at their preferred frequency while performing a secondary task (reaction times), but no significant effect of the dual task was found. The discrepancy of results between the two studies could be explained by the fact that the secondary task is not rhythmic in Aubin et al. (2021), whereas the secondary task implies a rhythmic component in Serrien (2009). This hypothesis is in accordance with the results of studies examining the effects of rhythmic external constraints (Bouvet et al., 2019, 2020). We could deduce that the SMT is robust to a general cognitive load but can be impacted by external rhythmic stimulation. Hence, we can recommend not to perform a rhythmic task before or during the production of a task assessing SMT because it can change the SMT values.

Regarding the external constraints, most studies (*N* = 5/7) report consistent results about the significant effects of external constraints, such as a noisy environment, the presence of fingertip contacts, or a varying spring constraint on the SMT values (Table 4). However, the effect of loading is not consistent (Hatsopoulos and Warren, 1996; Wagener and Colebatch, 1997; Hansen and Ohnstad, 2008).

The type of task seems to quasi-consistently modulate the SMT values in four out of five studies (Table 4). Specifically, results indicate that the SMT is affected by in-phase or anti-phase bimanual tapping, polyrhythmic or single rhythmic tapping, and by tapping, drawing, playing a melody, or reciting a sentence (Tajima and Choshi, 1999; Forrester and Whitall, 2000; Zelaznik et al., 2000; Pfordresher et al., 2021).

The internal state seems to modulate the SMT values as well (Table 4). Three out of 3 studies report an effect of the internal state, such as apnea, mental stress, and gravity on the SMT values (Murata et al., 1999; Dosseville and LaRue, 2002; Boulanger et al., 2020). Once again, these results indicate that the SMT is not robust and that intra-individual variability exists. In the same vein, the time of testing seems to have an effect on the SMT values (Table 4). More precisely, studies unanimously report an effect of the time of day on the SMT values (Oléron et al., 1970; Dosseville et al., 2002; Moussay et al., 2002; Wright and Palmer, 2020). It seems that the SMT values vary in the course of the day, being slower in the morning than in the evening (Moussay et al., 2002; Wright and Palmer, 2020). As for the effect of internal state mentioned above, this effect may be related to the circadian variations of internal physiological and psychological factors, such as hormones or fatigue. Anyway, it is important to interpret this result in relation to the results of many studies that have shown an effect of trial measurement (Collyer et al., 1994; Drake et al., 2000; Scheurich et al., 2018, 2020; Bouvet et al., 2019).

## 5. Conclusion and perspectives

All in all, our systematic review highlights large intra- and inter-individual variability in the SMT values. According to the internal clock model (Treisman, 1963), individuals have an internal clock that is a reference generating time information, used to perceive information, and to produce and reproduce behaviors. Each individual has his/her own internal clock, leading to strong intra-individual consistency, but individual preferences exist in the production and perception of rhythms. Moreover, the internal clock can be affected by many intrinsic and extrinsic factors. We hope that the current review will lead to a better choice of reference values for SMT. We have proposed specific recommendations and points of vigilance to assess the SMT in future research.

Our results could also be transferred to applied contexts related to rehabilitative, educative, and sport interventions involving rhythmic sensorimotor synchronization. For example, dance can be viewed as a rhythmic activity in which individuals have to learn a choreography in synchrony with rhythmic stimuli provided by music and partners. Irrespective of the context (e.g., rehabilitation, education, and sport), current studies recommend individualizing music-based rhythmic cueing to induce motor improvement (Dalla Bella et al., 2018). Given that performance in synchronization-continuation tasks is improved when the tempo of stimuli is closest to the SMT (Delevoye-Turrell et al., 2014) and that the SMT seems to predict performance in externally paced tasks such as sensorimotor synchronization (McPherson et al., 2018), the choice of the tempo of the music should be carefully determined to correspond to the SMT. However, our systematic review highlights that the SMT is not a fixed and universal value but rather a range of values, so it should be measured just before intervention to provide a reference at the time of the intervention, considering the effectors used to produce the task and the current conditions. Accordingly, the measurement of SMT should be explicitly and exhaustively described to interpret the value obtained (including the instructions provided to measure the SMT). To consider the large intra-individual variability of the SMT, we advise performing more than a single trial per participant to measure the SMT. In line with the recommendation of Amrani and Golumbic (2020), SMT consistency should be measured within a trial, within a session, and across sessions (Amrani and Golumbic, 2020). Finally, it could be interesting to conduct a similar systematic review on the preferred perceived tempo (PPT), which can be measured either as the chosen tempo among several tempi (Baruch et al., 2004; Bauer et al., 2015) or from a dynamic tempo adjustment (speed up or slow down) of a rhythmic metronome until individuals reach their preferred tempo (e.g., Amrani and Golumbic, 2020; Hine et al., 2022). Given the possible relationship between the SMT and the preferred music tempo (e.g., Hine et al., 2022), it is possible that a common tempo for motor and perceived preferences exists. In the case of a common internal clock, we could expect that similar factors affect the SMT and the PPT.

Interdisciplinary implications extend to the field of rehabilitative, educative, and sport interventions involving rhythmic sensorimotor synchronization. Indeed, studies have highlighted the strong role of rhythm in engagement, motivation, and pleasure in performing physical activities. In the context of sport performance, music—through its intrinsic qualities, such as rhythm and particularly its tempo—is known to promote engagement and involvement in a physical activity or sport (Karageorghis et al., 2021). For example, synchronization with music during endurance-based activities (treadmill running tasks) allows for increased time spent practicing (Terry et al., 2012). More globally, results from a meta-analytic review support “the use of music listening across a range of physical activities to promote more positive affective valence, enhance physical performance (i.e., ergogenic effect), reduce perceived exertion, and improve physiological efficiency” (Terry et al., 2020, p. 91).

As a conclusion, the present review provides new elements to understand the inter- and intra-variability of the SMT, and we hope that our recommendations will be taken into account in future studies investigating performance in rhythmic production and perception tasks.

## Author contributions

AD and JT primarily conducted this systematic review and wrote the first draft of the manuscript. EM provided expertise on the methodology for conducting a systematic review and participated in the discussions for the selection of articles. AD, EM, and JT collected all the information from the selected articles, provided feedback, and revised the manuscript. All authors contributed to the article and approved the submitted version.
